# Molecular Detection of Spotted Fever Group *Rickettsia* in Ticks Collected From Wildlife Carcasses at a Bushmeat Market in Ghana

**DOI:** 10.1002/puh2.70451

**Published:** 2026-11-07

**Authors:** Emmanuel Kwame Amoako, Seth Offei Addo, Richard Odoi‐Teye Malm, Caleb Kobina Danso‐Coffie, Elinam Adzo Agbobli, Rhoda Yeboah, Winnifred Offih‐Kyei, Eric Behene, Kofi Dadzie Kwofie, Sandra Abankwa Kwarteng, Langbong Bimi, Ben Adu Gyan, Daniel Oduro

**Affiliations:** ^1^ Department of Animal Biology and Conservation Science University of Ghana Accra Ghana; ^2^ Immunology Department Noguchi Memorial Institute for Medical Research University of Ghana Accra Ghana; ^3^ Parasitology Department Noguchi Memorial Institute for Medical Research University of Ghana Legon Ghana; ^4^ Department of Parasitology and Tropical Medicine Kitasato University School of Medicine Sagamihara Japan; ^5^ Department of Theoretical and Applied Biology College of Science Kwame Nkrumah University of Science and Technology Kumasi Ghana

**Keywords:** bushmeat, One Health, *Rickettsia africae*, *Rickettsia massiliae*, ticks, wildlife, zoonosis

## Abstract

Despite extensive bushmeat consumption in Ghana, data on tick‐borne zoonotic risks remain limited. This study investigated ticks collected from wildlife carcasses at the Atwemonom Bushmeat Market in Kumasi from April 2020 to April 2021 to assess the presence of zoonotic pathogens. Ticks were morphologically identified using standard taxonomic keys and screened for selected bacterial pathogens using PCR and Sanger sequencing. A total of 211 ticks were recovered from the 46 wildlife carcasses, with *Ixodes aulacodi* (49.76%) as the predominant species. Ticks were pooled (total pools = 94) by species and host; pool sizes ranged from 1 to 7 (Median 2). Rickettsial DNA was detected in 10.64% of tick pools, including *Rickettsia africae* and *Rickettsia massiliae*, whereas *Coxiella burnetii* was not detected. These findings highlight an underrecognized wildlife–human interface for potential rickettsial transmission and underscore the need for integrated surveillance and diagnostic capacity. This study establishes an essential epidemiological baseline that may inform future research and evidence‐based vector and disease management strategies.

## Graphical abstract

At the Atwemonom bushmeat market in Kumasi, 211 ticks were collected from 46 wildlife carcasses and grouped into 94 pools. *Rickettsia* DNA occurred in 10 pools (10.64%), comprising *Rickettsia africae* and *Rickettsia massiliae; Coxiella burnetii* was undetected. Findings establish an entomological baseline supporting integrated One Health surveillance without demonstrating pathogen transmission.

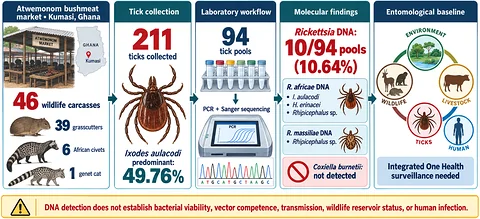

## 1 Introduction

Ticks are blood‐feeding ectoparasites, acting as vectors for a wide range of zoonotic infections [1, 2]. Ticks’ spread and growing habitats have increased human interaction with these vectors, allowing tick‐borne diseases (TBDs) to arise and re‐emerge across continents. Tick infections can spread to livestock and humans through wildlife hosts [3, 4]. This is especially true in areas where natural habitats are being impacted by human activity.

Wildlife in Sub‐Saharan Africa, including Ghana, serve as hosts for diverse tick species that transmit zoonotic pathogens such as *Rickettsia africae* and *Coxiella burnetii* [5, 6]. Wildlife‐livestock–human interfaces in the region often involve close interactions due to mixed land use and socioeconomic practices. Activities such as bushmeat hunting elevate the risk of zoonotic spillover [7, 8]. Tick‐borne pathogen transmission is influenced by complex ecological factors. Human‐driven environmental changes, including deforestation, agricultural expansion, and urbanization, disrupt wildlife habitats and shift tick distribution and host availability [9]. Climate change alters tick life cycles, survival rates, and host‐seeking behavior, expanding their range into new areas [10]. In Ghana and other West African countries, these environmental shifts intersect with traditional practices like bushmeat hunting and livestock farming. This combination increases human exposure to infected ticks and the potential for disease transmission [6].

Bushmeat markets are critical yet understudied points for zoonotic transmission, particularly during carcass handling and processing, which heighten human exposure to tick‐borne pathogens. Commonly traded species such as grasscutters, rats, antelopes, and other wild mammals often carry ticks that can transmit various infections [6, 11]. Hunters and traders face direct risk during skinning and processing, whereas residual ticks on meat can infest domestic animals or bite humans after sale, creating multiple pathways for pathogen spread. For example, *Ixodes aulacodi*, found on grasscutters in Ghana, is a known vector of bacterial and protozoan zoonoses [11]. Rising bushmeat demand, fueled by population growth, food insecurity, and cultural practices, compounds these risks, particularly in rural and peri‐urban areas with suboptimal hygiene [6, 12].

Recent studies in Ghana report diverse ticks and tick‐borne pathogens in livestock with documented pathogen exposure among abattoir workers [13, 14, 15, 16, 17, 18, 19, 20]. These findings highlight significant health risks and the persistence of transmission at the animal–human interface. Close interactions among humans, domestic animals and wildlife facilitate pathogen exchange, with ticks as key vectors. Surveys also reveal limited public awareness of TBDs, underscoring the need for education and community engagement [21, 22]. A One Health approach linking human, animal, and environmental health is essential to manage these zoonotic threats, emphasizing cross‐sector surveillance, risk assessment, and targeted interventions such as vector control, awareness campaigns, and informed policy development [23, 24].

Although *Rickettsia* has been found in ticks and livestock in Ghana and other West African nations [7, 25], little is known about *Rickettsia* in ticks that infest wildlife in Ghana. In particular, molecular data on *Rickettsia* in ticks collected from wildlife entering bushmeat markets is scarce. Bushmeat markets serve as an essential wildlife‐tick–human interface, bringing animal carcasses, ticks, hunters, traders, and butchers into close contact while also presenting an understudied context for entomological surveillance. Importantly, just because wildlife hosts ticks does not mean they function as pathogen reservoirs. Similarly, identifying *Rickettsia* DNA in ticks confirms the presence of pathogen‐associated genetic material but does not indicate bacterial viability, vector competence, transmission, or reservoir competence [26, 27]. This study aimed to identify ticks collected from wildlife carcasses in a Ghanaian bushmeat market and determine the presence of *Rickettsia* DNA using molecular methods, providing baseline data for future integrated investigations at the wildlife–human interface.

## 2 Methods

### 2.1 Study Area

The research was carried out at the Atwemonom bushmeat market in Kumasi, Ghana's second‐biggest city, which is in the Ashanti region. Atwemonom is one of Kumasi's largest and most established formal markets, specializes in fresh bushmeat, and serves as a key trading hub for surrounding regions, offering insights into local bushmeat trade dynamics (Figure 1). The market is a vital source of protein for city dwellers and revenue for vendors, and it is situated close to a major road in the heart of Kumasi. Strategically situated along a major roadway in central Kumasi, Atwemonom provides an ideal setting for investigating wildlife‐associated ticks within the urban bushmeat trade [12].

**FIGURE 1:**
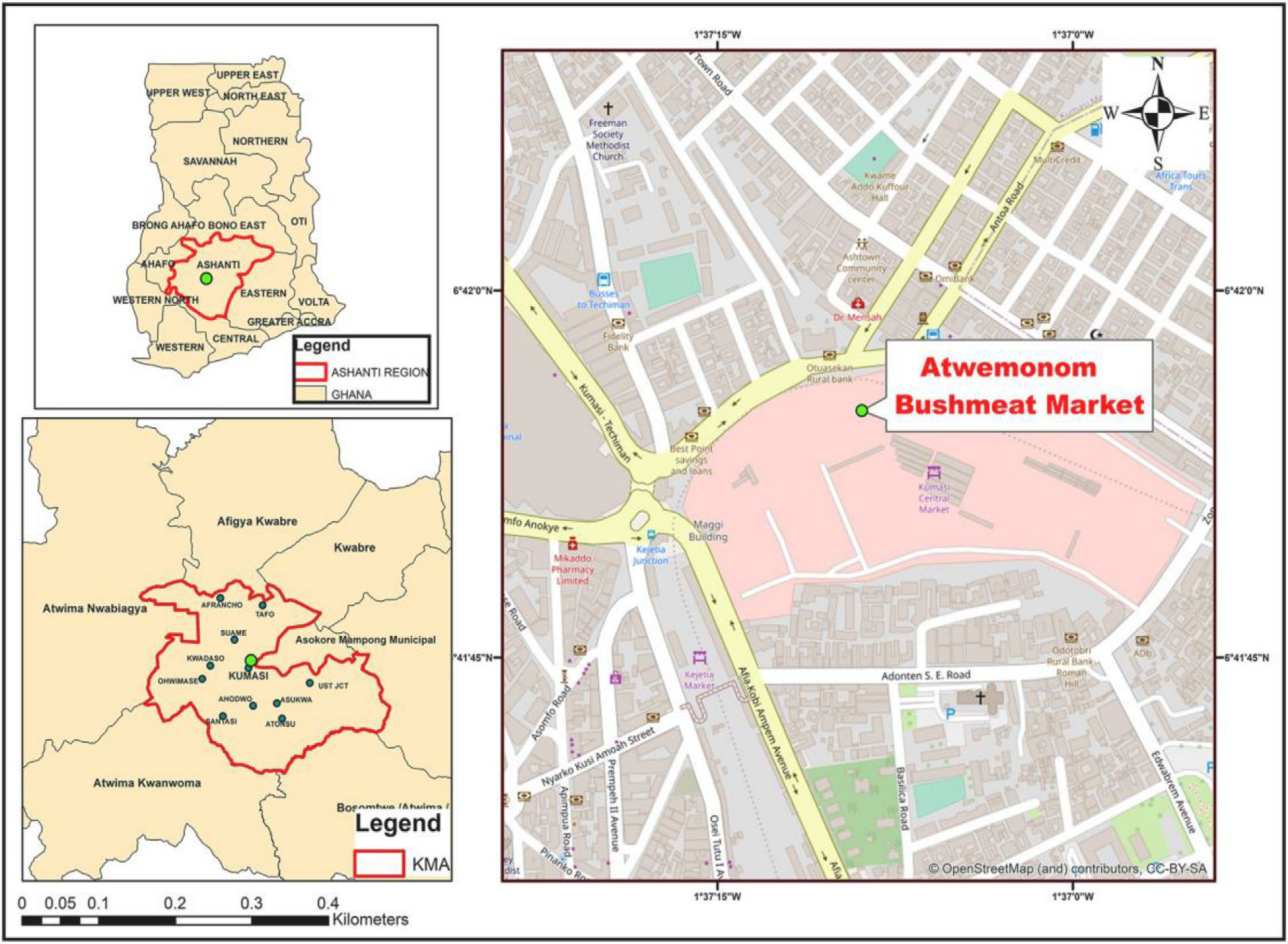
Map showing the study site, Atwemonom (bushmeat market), and wild animal hunting districts in Ghana.

### 2.2 Tick Sampling

Ticks were conveniently collected from fresh wild animal carcasses displayed at the bushmeat market between April 2020 and April 2021. The carcasses were selected on the basis of availability. The hunters reported that the carcasses were from different locations in the Ashanti and Bono regions. However, the precise geographic origins could not always be independently verified. Every carcass was carefully inspected for tick attachments, paying particular attention to the ventral surfaces, ears, neck, and groin. To guarantee entire extraction, minimize the possibility of damaging specimens, and maximize the quality of samples for identification, ticks were meticulously removed using blunt forceps. Collected ticks were placed in labeled vials containing 70% ethanol and transported to the laboratory for morphological identification using standard keys [28, 29]. The features used for identification included characters of the capitulum, basis capituli, palps, hypostome, scutum, festoons, spiracular plates, coxae, genital aperture, and overall body morphology, as appropriate for the sex and developmental stage of the specimens. Molecular confirmation was not conducted, and voucher specimens were not deposited, so potential misidentification, particularly among morphologically similar *Rhipicephalus* taxa, cannot be totally ruled out. Each specimen's metadata, such as the host species, collection date, and location, were documented. Sampling was conducted on market days under permission from market authorities; no live animals were handled. Seasonal variation was not analyzed, but the sampling spanned both dry and wet seasons.

### 2.3 Molecular Detection of Tick‐Borne Pathogens

Individual ticks were surface‐decontaminated by washing them in 70% ethanol and sterile molecular‐grade water to remove any exterior pollutants. The ticks were then air‐dried in sterile conditions. The ticks (entire ticks) were pooled (1–7 per pool) on the basis of host species, tick species, and tick sex. Pooling is widely used in tick surveillance to estimate pathogen prevalence under resource constraints and is appropriate for low‐prevalence pathogens [30]. The tick pools were homogenized in a Mini‐Beadbeater‐96 (BioSpec, Bartlesville, OK, USA), and total nucleic acid was extracted using the QIAamp Viral Extraction Kit (QIAGEN, Valencia, CA, USA) in accordance with the manufacturer's instructions.

Using the primers Trans‐1 (TATGTATCCACCGTAGCCAGTC) and Trans‐2 (CCCAACAACACCTCCTTATTC), *C. burnetii* was detected by targeting the IS1111 fragment, a transposon‐like repetitive region, amplifying at 687 bp [31, 32]. The PCR reaction consisted of 25 µL of 2x GoTaq Hot Start Green Master Mix (Thermo Fisher Scientific), 18 µL of nuclease‐free water, 1 µL of each primer (10 µM stock solution), and 5 µL of the DNA template. Using a Mastercycler X50 PCR thermocycler (Eppendorf, Germany), the cycling conditions were initial denaturation at 95°C for 2 min; 5 touchdown cycles of 94°C for 30 s, annealing starting at 66°C and decreasing by 1°C per cycle to 62°C for 1 min, and 72°C for 1 min; followed by 35 cycles of 94°C for 30 s, 61°C for 30 s, and 72°C for 1 min; with a final extension at 72°C for 10 min and hold at 4°C. The positive control was *C. burnetii* DNA, whereas the negative control was nuclease‐free water.

To detect *Rickettsia* DNA in tick pools, a 632‐bp amplification was accomplished using primers 190‐70F (ATGGCGAATATTTCTCCAAAA) and 190‐701R (GTTCCGTTAATGGCAGCATCT) that target the *ompA* of *Rickettsia* [33]. PCR reaction conditions were identical to those described above. The cycling conditions were 95°C for 5 min, 34 cycles at 95°C for 30 s, 51.5°C for 30 s, 72°C for 1 min, and a final extension of 72°C for 5 min. The run was held at 4°C. The positive control was *R. africae* DNA, whereas the negative control was nuclease‐free water. Following separation of the PCR products from the conventional PCRs on a 2% agarose gel and visualization with a Molecular Imager Gel Doc, positive pools showing the expected band size were shipped to Macrogen Europe B.V. for purification and sequencing. They were sequenced bidirectionally using Sanger sequencing.

### 2.4 Data Analysis

STATA version 13 was used for the statistical analysis, and *p* < 0.05 was chosen as the significance level. Associations between rickettsial pathogen detection and host‐related variables were assessed using a generalized linear mixed‐effects model (GLMM) with a binomial error distribution, with sample ID included as a random effect to account for clustering among observations. The PooledBin function from the PooledInfRate package in R version 4.3.3 was used to generate infection rates. Individual‐level infection probabilities were estimated using Firth's bias‐reduced maximum likelihood method, with 95% confidence intervals calculated using the skewness‐corrected score method, accounting for the number and size of tick pools. The sequences obtained in this study were viewed using Chromas (version 2.6.6) and edited and cleaned, and a consensus sequence was created in MEGA (version 10.0.5). The consensus sequences were aligned with other sequences in the NCBI database. The sequences were aligned within MEGA (version 10.0.5) using the Clustal Omega tool [34]. Using the best fit model (GTR + G + I) selected by BIC, the aligned sequences were then used to generate phylogenetic trees using the maximum likelihood method inferred with 1000 bootstrap replicates. The country of origin and accession numbers for the GenBank sequences utilized in the phylogenetic analysis have been indicated.

### 2.5 Ethical Considerations

The study involved sampling wild animal carcasses obtained from licensed hunters and traders. Approval to access these licensed hunters and traders was granted by the Wildlife Division of the Forestry Commission of Ghana and the Bushmeat Market Management, with additional permission provided by the Market Queen of the Bushmeat Traders Association. No live animals were examined or involved in the study.

## 3 Results

During the sampling period, a total of 46 wild animal carcasses were examined for tick collection. Of these, 34 were hunted from various locations within the Ashanti region, and 12 were from neighboring regions (Figure 1). The wild animals assessed included 39 grass cutters, 1 genet cat, and 6 African civets. Grass cutters accounted for 84.8% of sampled hosts, reflecting market availability. From the collected samples, a total of 211 ticks were identified, comprising 153 females and 58 males. The predominant species observed was *I. aulacodi*, accounting for 49.76% of the total tick population (Figure 2). Notably, grass cutters exhibited a higher incidence of tick species infestation compared to the genet and African civets (Figure 3).

**FIGURE 2:**
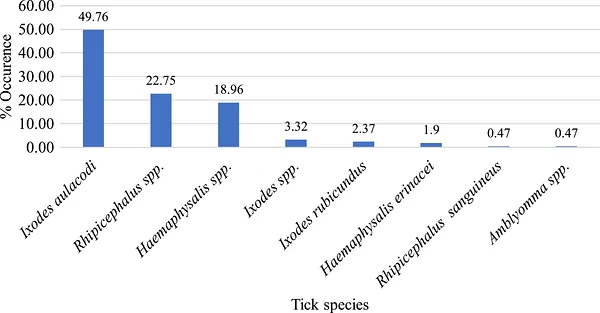
Distribution of tick species identified.

**FIGURE 3:**
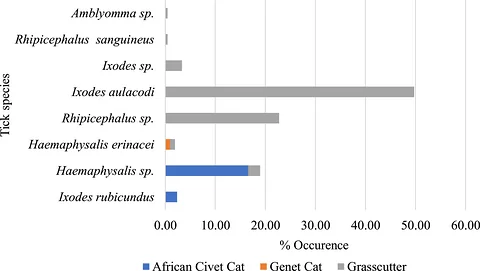
Diversity of tick species infesting sampled wildlife.

### 3.1 Molecular Detection of Tick‐Borne Pathogens in Sampled Ticks

From the 94 tick pools screened, 10 (10.64%) were positive for *Rickettsia* DNA by standard PCR of the *ompA* gene with identification based on amplicon size. The *Rickettsia* DNA was detected in pools of *I. aulacodi* (4.26%), *Haemaphysalis erinacei* (1.06%), and *Rhipicephalus* sp. (5.32%). None of the pools was positive for *C. burnetii*. OmpA amplicon sequencing identified two distinct *Rickettsia* species in the positive pools, following the speciation of all *Rickettsia* PCR‐positive samples. *R. africae* was identified in 4 (11.11%) *I. aulacodi*, 1 (50%) *H. erinacei* and 3 (9.38%) *Rhipicephalus* sp. pools. *Rickettsia massiliae* was detected in 2 (6.25%) *Rhipicephalus* sp. pools. The pool‐level dataset is shown in Table S1. The highest maximum likelihood estimation (MLE) rate of *R. africae* was recorded as 25.46% (95% CI: 1.64, 81.95%) in pools of *H. erinacei* (Table 1). The MLE of *R. massiliae* was recorded as 4.22% (95% CI: 0.76, 13.18%) in pools of *Rhipicephalus* sp. No significant associations were observed between the identified *Rickettsia* species and characteristics such as host type or tick sex (Table 2).

**TABLE 1:** Distribution of *Rickettsia* detections by tick species at the Atwemonom market.

Tick species	Pathogen	Number of ticks	Number of positive tick pools	Infection rate (95% CI)^a^
*Haemaphysalis erinacei*	*Rickettsia africae*	4	1	25.46 (1.64–81.95)
*Ixodes aulacodi*	*Rickettsia africae*	105	4	3.93 (1.30–9.16)
*Rhipicephalus* spp.	*Rickettsia africae*	48	3	6.34 (1.70–16.17)
	*Rickettsia massiliae*		2	4.22 (0.76–13.18)
*Amblyomma* sp.	—	1	0	—
*Haemaphysalis* spp.	—	40	0	—
*Ixodes oldi*	—	5	0	—
*Ixodes* sp.	—	7	0	—
*Rhipicephalus sanguineus*	—	1	0	—

^a^ Infection rate was calculated using the maximum likelihood estimation method.

**TABLE 2:** Association between *Rickettsia* species, host, and tick sex.

		Pooled positive, *n* (%)			
	No. of tick pools	*Rickettsia africae*	*p* value^a^	*Rickettsia massiliae*	*p* value^a^
Animal host					
African civet cat	17	0 (0.0)	0.679	0 (0.0)	0.952
Genet cat	1	0 (0.0)		0 (0.0)	
Grass cutter	76	8(10.5)		2 (2.6)	
Tick sex					
Male	58	6 (10.3)	0.610	2 (3.5)	0.777
Female	36	2 (5.6)		0 (0.0)	
Total	94	8 (8.51)		2 (2.13)	

^a^ *p* value was obtained using a generalized linear‐effect mixed model.

Nine reference sequences retrieved from the NCBI GenBank database were selected on the basis of their relevance to the *Rickettsia* taxa represented in the study, availability of comparable *ompA* sequences, and suitability for phylogenetic comparison. On the basis of the phylogenetic analysis, *R. africae* from this study was related to isolates from Benin (KT633262), Ghana (OQ331038), and South Africa (OQ130421) with a bootstrap value of 86% (Figure 4). Again, *R. massiliae* from this study were related to isolates from China (PP104812 and MF098409) with a bootstrap support of 99%.

**FIGURE 4:**
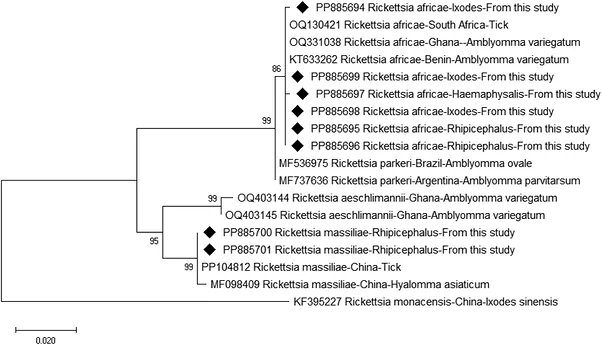
Maximum likelihood phylogenetic tree inferred from the *ompA* gene sequences generated in this study and representative *Rickettsia* sequences retrieved from GenBank. The analysis was based on an alignment of 596 nucleotide positions using the GTR + G + I substitution model selected according to the Bayesian Information Criterion (BIC), with 1000 bootstrap replicates. Bootstrap values ≥50% are shown at the corresponding nodes. Sequences generated in this study and GenBank reference sequences are identified by their accession numbers. *Rickettsia monacensis* was used as the outgroup for tree rooting.

The sequences from this study have been deposited in GenBank: *R. africae* (Accession numbers: PP885694, PP885695, PP885696, PP885697, PP885698, and PP885699) and *R. massiliae* (Accession numbers: PP885700 and PP885701).

## 4 Discussion

Wildlife hunted as bushmeat in Ghana were found infested with different tick species. It was observed that grass cutters, commonly hunted and consumed, were infested with diverse tick species, indicating their role in the spread of ticks and potentially infectious pathogens. Furthermore, *I. aulacodi*, infesting only grass cutters, was the predominant tick species, a finding comparable to a previous study in Ghana [11]. This study reports the first molecular detection of *R. massiliae* and *R. africae* in ticks infesting wildlife in Ghana. The findings indicate the presence of *Rickettsia*‐associated DNA in tick samples but do not establish bacterial viability, vector competence, or transmission potential.

### 4.1 Ecological and Epidemiological Insights

The detection of *R. africae* in *I. aulacodi*, *H. erinacei*, and *Rhipicephalus* species infesting grass cutters confirms that rickettsial pathogens are associated with wildlife [25]. The current study had insufficient statistical power to reliably say whether wildlife hosts act as reservoirs capable of sustaining these organisms in nature, given the small and unbalanced host sample sizes. However, as grass cutters are frequently targeted for bushmeat, they serve as a direct conduit between human populations and animals, which raises the possibility of tick contact. Hunters, traders, butchers, and anyone that handles and processes wildlife may be exposed to ticks that carry *Rickettsia*‐associated DNA. The current study, however, did not look at human exposure or infection; hence, there is no clear link between infected ticks from wildlife carcasses and human rickettsioses. More research, including genetic and serological surveillance in exposed human populations, domestic animals, wildlife, and questing or host‐associated ticks, is needed to identify whether transmission takes place across these interfaces. The detection of *R. massiliae* and *R. africae* DNA adds to the baseline understanding of rickettsial diversity in ticks linked with wildlife in Ghana. Although both organisms have been linked to human infections [35, 36, 37, 38], their presence in ticks in this study does not indicate the presence of human rickettsiosis in the area. Rather, these findings suggest a feasible location for future research using combined vector, animal, and human surveillance.

### 4.2 Public Health Significance

The detection of *Rickettsia* DNA in ticks collected from wildlife carcasses has potential public health implications because humans who hunt, handle carcasses, butcher, and process bushmeat may come into contact with ticks. However, the study did not analyze human infection or exposure; therefore, the associated health risk cannot be quantified. The symptoms of *R. africae*‐caused African tick‐bite fever frequently include fever, headache, rash, and eschar at the site of the tick bite [37]. *R. massiliae* has also been associated with human rickettsiosis in other settings [35, 36, 38].

These documented connections provide a foundation for additional research, but they should not be interpreted as proof that human rickettsial disease is now emerging in the study area. As a result, the findings indicate the importance of ongoing entomological surveillance and occupational knowledge among those who handle wildlife and carcasses on a regular basis. Future research should focus on human exposure, including serological evidence of infection, and ticks from animals, livestock, and the environment to determine whether the observed *Rickettsia* are circulating across these interfaces.

### 4.3 One Health Perspective

A One Health framework provides a solid foundation for examining the possible implications of *Rickettsia* DNA found in wildlife‐associated ticks. The current findings provide an entomological baseline, but they do not indicate independent transmission between wildlife, ticks, livestock, and humans. Integrated surveillance encompassing wildlife hosts, livestock, ticks, humans, and relevant environmental settings is thus required to evaluate whether the observed rickettsiae persist locally and whether transmission happens across ecological interfaces. Such studies could integrate molecular characterization of *Rickettsia* in tick and animal materials with human serological and clinical surveillance. Hunting, carcass processing, bushmeat trading, livestock rearing, and veterinary activities could all benefit from additional consideration. Public education on tick avoidance and careful management of wildlife carcasses may also be acceptable as precautionary interventions, albeit their efficiency should be assessed in light of the local epidemiological setting.

Regional collaboration and the exchange of genomic and epidemiological data may increase our understanding of the geographic distribution and variety of tick‐associated rickettsiae in West Africa. Importantly, surveillance findings should be combined with evidence from human and animal populations before concluding zoonotic transmission or disease onset.

### 4.4 Limitations and Future Directions

Some limitations should be considered when interpreting these results. First, tick identification relied on morphological criteria rather than molecular confirmation, which may have led to misidentification of morphologically identical taxa. Second, the small and uneven number of wildlife hosts studied, along with market‐based sampling, may limit the findings’ representativeness and generalizability. Third, no human exposure data were collected; therefore, the potential public health relevance of the identified *Rickettsia* cannot be determined directly. Furthermore, the identification of *Rickettsia* DNA in ticks does not indicate bacterial survival, vector competence, or transmission potential. Finally, pooled PCR analysis may have lower sensitivity for infected ticks with low pathogen loads and does not allow for the assessment of infection status at the individual tick level. Future studies should incorporate enhanced metagenomic sequencing and longitudinal surveillance across diverse ecological zones to identify both existing and emerging pathogens. Improved understanding of spillover mechanisms achieved through investigation of infection patterns in humans and domestic animals coexisting with wild animals will inform the development of comprehensive One Health interventions, supported by concurrent human serological data.

## 5 Conclusion

This study reports the molecular detection of *R. massiliae* and *R. africae* in wildlife ticks in Ghana. These findings add to our understanding of rickettsial diversity in wildlife‐associated ticks, but they do not establish bacterial viability, vector competence, transmission, or reservoir role for wildlife hosts. Given the possible occupational interaction between wildlife, ticks, and humans, more integrated surveillance of ticks, wildlife, livestock, and exposed human populations is needed to establish the epidemiological significance of these detections. Continued molecular characterization and longitudinal studies will be critical in determining the prevalence, diversity, and potential transmission mechanisms of tick‐associated *Rickettsia* in Ghana and the wider West African region.

## Author Contributions

**Emmanuel Kwame Amoako**: investigation, formal analysis, methodology, data curation, validation, writing – original draft, software. **Seth Offei Addo**: formal analysis, data curation, methodology, validation, writing – original draft, software. **Richard Odoi‐Teye Malm**: data curation, investigation. **Caleb Kobina Danso‐Coffie**: methodology, investigation, data curation. **Elinam Adzo Agbobli**: methodology, investigation. **Rhoda Yeboah**: investigation, methodology. **Winnifred Offih‐Kyei**: methodology, investigation. **Eric Behene**: formal analysis, data curation. **Kofi Dadzie Kwofie**: formal analysis, investigation. **Sandra Abankwa Kwarteng**: formal analysis, supervision, writing – review and editing. **Langbong Bimi**: supervision, writing – review and editing, visualization, funding acquisition. **Ben Adu Gyan**: supervision, writing – review and editing. **Daniel Oduro**: conceptualization, methodology, investigation, formal analysis, supervision, funding acquisition, project administration, resources, writing – review and editing.

## Funding

This study was partly supported by the University of Ghana through internal research funding.

## Conflicts of Interest

The authors declare no conflicts of interest.

## Supporting information




**Table S1**: Surveillance data on tick collections, including study location, host species, tick identification, molecular results, and Genebank accession numbers.

## Data Availability

The data supporting this study are included in the article.
